# Strengthening India’s trauma system: consolidating progress into accountable systems

**DOI:** 10.1136/tsaco-2025-001953

**Published:** 2025-06-26

**Authors:** Divya Kewalramani, Mayur Narayan

**Affiliations:** 1 Department of Surgery, Rutgers Robert Wood Johnson Medical School, New Brunswick, New Jersey, USA

**Keywords:** Multiple Trauma, health policy, education, Emergency Medical Services

 The review by James *et al* offers a focused perspective of India’s trauma ecosystem.^1^ Several new developments across the trauma-care continuum merit additional consideration.

## Primary injury prevention

India’s adaptation of the Vision Zero ethos is exemplified by the NGO-led Zero Fatality Corridor (ZFC) on the Mumbai–Pune Expressway, an initiative that has reduced crash deaths by 58% since 2016.^2^ Encouraged by these results, ZFC has been extended to 100 additional national highways, demonstrating how public-private partnerships can translate a safety framework into measurable public health gains.

## Prehospital response

India’s nascent prehospital care system remains fragmented, making bystander interventions critically important. Despite the significant progress driven by the Good Samaritan Law,^3^ prehospital care continues to face challenges due to the existence of over 20 emergency numbers nationwide, causing critical delays, compounded by inconsistent ambulance services. Implementing a unified emergency number and enhancing ambulance networks would substantially reduce preventable mortality. The recent introduction of India’s first helicopter emergency medical services in Uttarakhand provides a promising blueprint for efficient trauma response nationwide.^4^

## Hospital care

Excess in-hospital mortality stems primarily from disorganization that breaches critical golden-hour principles, rather than technological limitations. Many hospitals triage medical and surgical emergencies together, lacking dedicated trauma bays, 24/7 trauma teams, and after-hours imaging. Consequently, these centers report mortality rates (21%) twice that of high-resource settings (10%) for comparable traumatic injuries.^5^ The solution lies not only in additional accreditation but also in systemization: clear trauma triage lanes, prearrival ambulance alerts, minimum imaging standards, and mandatory basic life support and trauma evaluation and management (TEAM) training for every cadre that touches a trauma patient.^6^

## Training and workforce

Despite the expansion of advanced trauma life support programs targeted primarily at specialist physicians, basic trauma care skills for bystanders and frontline nursing staff remain significantly underemphasized. For instance, a multicenter pilot demonstrated that a brief *Stop the Bleed* training curriculum triples correct tourniquet placement among participants.^7^

## System governance

To date, the Indian government has funded 196 hospitals as graded trauma care facilities; however, performance remains inconsistent without enforceable standards, equipment, and data reporting. Given India’s substantial population and the enormous morbidity and mortality burden from trauma, enshrining a ‘Right to Trauma Care’ in national legislation could standardize care, mandate appropriate resource allocation, and reinforce accountability. It could serve as a powerful foundation for creating a cohesive, equitable, and effective trauma care system nationwide.
